# Additional Normothermic Machine Perfusion Versus Hypothermic Machine Perfusion in Suboptimal Donor Kidney Transplantation: Protocol of a Randomized, Controlled, Open-Label Trial

**DOI:** 10.29337/ijsp.165

**Published:** 2021-10-06

**Authors:** Elsaline Rijkse, Sarah Bouari, Hendrikus J. A. N. Kimenai, Jeroen de Jonge, Ron W. F. de Bruin, Julia S. Slagter, Martijn W. F. van den Hoogen, Jan N. M. IJzermans, Martin J. Hoogduijn, Robert C. Minnee

**Affiliations:** 1 Erasmus MC Transplant Institute, Division of HPB and Transplant Surgery, Department of Surgery, Erasmus MC University Medical Center, Rotterdam, the Netherlands; 2 Erasmus MC Transplant Institute, Nephrology and Transplantation, Department of Internal Medicine, Erasmus MC University Medical Center, Rotterdam, the Netherlands

**Keywords:** Kidney transplantation, Organ preservation/methods, perfusion, randomized controlled trial

## Abstract

**Introduction:**

Ageing of the general population has led to an increase in the use of suboptimal kidneys from expanded criteria donation after brain death (ECD-DBD) and donation after circulatory death (DCD) donors. However, these kidneys have inferior graft outcomes and lower rates of immediate function. Normothermic machine perfusion (NMP) may improve outcomes of these suboptimal donor kidneys. Previous non-randomized studies have shown the safety of this technique and suggested its efficacy in improving the proportion of immediate functioning kidneys compared to static cold storage (SCS). However, its additional value to hypothermic machine perfusion (HMP), which has already been proved superior to SCS, has not yet been established.

**Methods and analysis:**

This single-center, open-label, randomized controlled trial aims to assess immediate kidney function after 120 minutes additional, end-ischemic NMP compared to HMP alone. Immediate kidney function is defined as no dialysis treatment in the first week after transplant. Eighty recipients on dialysis at the time of transplant who receive an ECD-DBD or DCD kidney graft are eligible for inclusion. In the NMP group, the donor kidney is taken of HMP upon arrival in the recipient hospital and thereafter put on NMP for 120 minutes at 37 degrees Celsius followed by transplantation. In the control group, donor kidneys stay on HMP until transplantation. The primary outcome is immediate kidney function.

**Ethics and dissemination:**

The protocol has been approved by the Medical Ethical Committee of Erasmus Medical Center (2020-0366). Results of this study will be submitted to peer-reviewed journals.

**Registration:**

registered in *clinicaltrials.gov* (NCT04882254).

**Highlights:**

- This is the first RCT to compare additional NMP to HMP alone.
- Extensive sampling will offer in-depth analysis of kidney physiology during NMP.
- This RCT may help identify biomarkers to predict clinical outcomes during NMP.
- Biomarkers can help develop NMP as assessment tool for declined kidneys.

## 1. Background

The main concern for patients waiting on a kidney transplant is the large gap between the demand and supply of suitable donor kidneys. Consequently, patients are on average waitlisted for three to four years while experiencing inferior quality of life and decreased survival compared to transplanted patients [1,2]. Ageing of the general population has led to an increase in the use of suboptimal kidneys from expanded criteria donation after brain death (ECD-DBD) donors, defined as a DBD donor of 60 years or older or 50–59 years with cardiovascular comorbidities, and donation after circulatory death (DCD) donors [3,4,5]. Kidneys of these donors are considered of lower quality compared to standard criteria DBD donors due to a higher prevalence of comorbidities or, in case of DCD donation, an additional period of warm ischemic time. As a result, the transplantation rate of procured ECD-DBD and DCD kidneys is much lower compared to standard criteria DBD kidneys [5].

Kidneys from an ECD-DBD donor have a 70% higher risk of graft failure and a higher risk of acute rejection and delayed graft function (DGF) compared to standard criteria DBD kidneys [3,6]. DCD kidneys have an increased risk of graft loss within 90 days, primary non-function (PNF) and DGF [3]. DGF, defined as the need for dialysis in the first week after transplant, is associated with a shorter graft survival, higher rates of acute cellular rejection, an increased length of hospital stay and increased costs [7,8,9]. The mechanism underlying the association between DGF and inferior graft outcomes is thought to be related to ischemia-reperfusion injury with ischemia from a variety of donor characteristics superimposed upon immune and inflammatory responses during reperfusion, resulting in acute tubular necrosis [10,11]. Because DGF is an early marker of graft function, it is often used as a surrogate endpoint in clinical studies regarding kidney transplantation.

Transplantation of these suboptimal kidneys necessitates the development of other preservation techniques to improve graft outcomes. Donor organs are currently preserved on static cold storage (SCS) or hypothermic machine perfusion (HMP) to minimize ischemic damage and reduce metabolism and oxygen demand [12]. However, because of this reduced metabolism, the capacity for tissue repair is limited. Furthermore, toxic substances, such as adenosine, inosine and hypoxanthine, accumulate in the cell [13]. As a result, each additional hour of cold ischemia leads to an increase in the risk of graft failure [14]. During normothermic machine perfusion (NMP), the kidney has full metabolic activity, allowing initiation of repair processes and restoration of adenosine triphosphate (ATP) levels under controlled conditions without the immune components present in the recipient [13]. Experimental studies have already shown improved kidney function, reduced tubular injury and increased expression of heat shock proteins suggesting upregulation of conditioning mechanisms compared to cold preservation strategies [15,16,17]. Even though animal studies suggest superiority of longer periods of NMP, full replenishment of ATP has already been demonstrated after 120 minutes [18,19].

So far, no randomized controlled trial (RCT) has been performed to establish these theoretical advantages in clinical practice. Clues about efficacy are found in cohort comparisons from two clinical studies carried out in three centers in the United Kingdom and Rotterdam, showing a clinically relevant reduction in the proportion of patients with DGF [20,21,22]. Currently, an RCT is carried out in the United Kingdom to assess the efficacy of 60 minutes additional, end-ischemic NMP compared to SCS only [23]. A previous RCT found that HMP kidneys had less DGF and improved graft survival in the first year after transplant compared to SCS [24]. This has led to a protocol change in the Netherlands, meaning that transportation of deceased donor kidneys on HMP is standard of care. Moreover, all kidney grafts from DCD donors aged 50 years or older are transported using oxygenated HMP following results from the COMPARE trial [25]. No RCT has been initiated yet to assess the efficacy of end-ischemic NMP in addition to HMP. This comparison is important, as HMP is safer (failure of NMP leads to additional warm ischemic time), less time-consuming, cheaper, and easier to carry out. Therefore, our present, single-center RCT aims to investigate the efficacy of 120 minutes additional, end-ischemic NMP in comparison to HMP alone on immediate graft function after ECD-DBD and DCD kidney transplantation.

## 2. Methods and analysis

**Figure 1:**
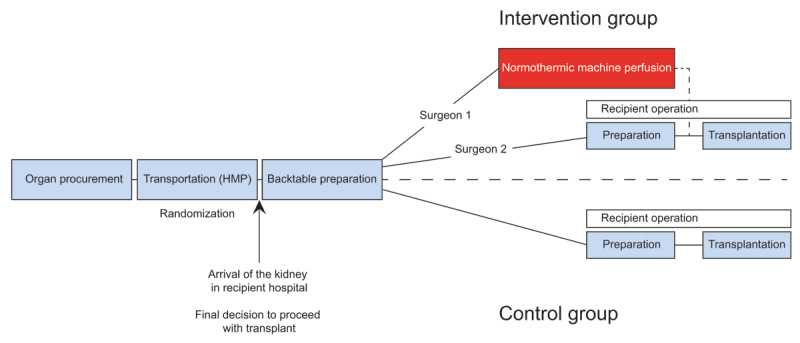
Study procedure for the intervention group and control group.

This study (acronym: APOLLO study) is designed as a randomized, controlled, single-center, open-label trial in 80 patients undergoing ECD-DBD or DCD kidney transplantation. Kidney grafts in the intervention group are transported on HMP followed by 120 minutes of end-ischemic NMP and kidney transplantation. Kidney grafts in the control group are transported and kept on HMP until transplantation. The flow chart of the study procedure is presented in ***Figure 1***.

### 2.1 Study endpoints

The primary endpoint is immediate graft function. This outcome is defined as no need for dialysis in the first week after transplant irrespective of recovering graft function.

The secondary endpoints are the following (summarized in ***Table 1***):

1. The incidence of DGF. DGF is defined as the need for dialysis in the first week after transplantation with recovering graft function, including dialysis for hyperkalemia or volume overload.
2. The incidence of DGF, excluding dialysis sessions for hyperkalemia or volume overload.
3. Duration of DGF, which is defined as the time between transplant and the last dialysis session.
4. Total number of post-transplant dialysis sessions.
5. The incidence of PNF. PNF is defined as a never functioning graft and is concluded retrospectively after 3 months.
6. Estimated glomerular filtration rate (eGFR) trajectory in the first year after transplant calculated with the Chronic Kidney Disease Epidemiology collaboration (CKD-EPI) formula.
7. eGFR at 1 year, 3 years and 5 years calculated with the CKD-EPI formula.
8. Biopsy-proven acute rejection within the first year post-transplant.
9. All-cause and death-censored graft survival up to 5 years. Uncensored graft survival counts death with functioning graft as event. For death-censored graft survival, patients are censored if they die with a functioning graft.
10. Patient survival.
11. Length of hospital stay, calculated from transplantation date until the date of discharge.
12. The incidence and severity of (serious) adverse events graded according to the Common Terminology Criteria for Adverse Events (CTCAE, version 4.0).
13. Postoperative complications, graded according to the Clavien-Dindo classification [26].
14. Ischemia-reperfusion injury determined by serum levels of donor-derived cell-free DNA, measured at day 1–6 in the patient [27]. Day 0 is defined as the interval between arterial reperfusion and the midnight of that day.
15. Histology and gene expression analysis of kidney biopsies during the course of NMP and compared with reperfusion biopsies in the control group.

**Table 1:** Endpoints of the study.


**Primary endpoint**

**1.** Immediate graft function

**Secondary endpoints**

**2.** DGF

**3.** DGF, excluding hyperkalaemia and volume overload

**4.** Duration of DGF

**5.** Total number of post-transplant dialysis sessions

**6.** PNF

**7.** eGFR trajectory

**8.** eGFR at 1 year, 3 years, 5 years

**9.** Biopsy-proven acute rejection ≤1 year

**10.** All-cause/death-censored graft survival

**11.** Patient survival

**12.** Length of hospital stay

**13.** (serious) adverse events

**14.** Postoperative complications

**15.** Quantity of donor-derived cell-free DNA

**16.** Renal histology/gene expression during NMP compared with HMP reperfusion biopsies

**NMP group only**

**17.** Perfusion dynamics (renal blood flow, intrarenal resistance)

**18.** Microcirculation and oxygenation

**19.** Markers of kidney damage/function

**20.** Perfusate sodium/urine sodium ratio

**21.** Oxygen consumption

**22.** Urine production

**23.** Quantity/composition of extracellular vesicles

**24.** Determination of the Hosgood score

The following endpoints are assessed only in the NMP kidneys. The goal is to gain a better understanding of kidney physiology during NMP and to assess whether measurements during NMP are correlated with clinical outcomes. Time points of these measurements are summarized in ***Table 2***:

1. Renal blood flow, intrarenal resistance and pressure during NMP, which is measured continuously.
2. Microcirculation and oxygenation during NMP measured with the MoorO2Flo laser speckle camera. These measurements are performed at three predefined timepoints.
3. Perfusate analysis for markers of kidney damage and function during the course of NMP.
4. Perfusate and urine sample analysis to determine the perfusate sodium/urine sodium ratio during the course of NMP.
5. Blood gas analysis with calculation of the oxygen consumption of the kidney during the course of NMP.
6. Urine production.
7. Measurements of the quantity and composition of extracellular vesicles during NMP [28].
8. Determination of the Hosgood assessment score at start of NMP, after 60 minutes and after 120 minutes [29]. This score is only registered and not used to determine suitability of the graft.

**Table 2:** Timing of measurements and sample collection during NMP.


	0 min	15 min	30 min	45 min	60 min	75 min	90 min	105 min	120 min

**Measurements**									

Renal blood flow/intrarenal resistance/pressure	Continuously								

Kidney weight (in grams)	X	–	–	–	–	–	–	–	X

Microcirculation/oxygenation	–	X	–	–	–	X	–	X	–

Arterial and venous blood gas	X	–	X	–	X	–	X	–	X

Urine production	–	–	X	–	X	–	X	–	X

Hosgood assessment score	X	–	–	–	X	–	–	–	X

**Samples**									

Perfusate	X	–	X	–	X	–	X	–	X

Urine	–	–	X	–	X	–	X	–	X

Biopsy	X	–	–	–	X	–	–	–	X

### 2.2 In- and exclusion criteria

All eligibility criteria are summarized in ***Table 3***. Patients aged 18 years or older who provide written informed consent and receive an ECD-DBD or DCD Maastricht type III-V kidney are eligible to participate in the study if they meet the following inclusion criteria: kidney-only transplant, on hemodialysis or peritoneal dialysis at time of transplant and standard immunosuppression regimen. Patients are not eligible for inclusion if they are pre-emptive at time of transplant, receive a multi-organ or dual kidney transplant, or if the age of the donor or recipient is below 18 years of age. Highly immunized patients with virtual panel reactive antibodies equal to or higher than 85% are excluded, as well as patients for whom it is agreed in advance that they need post-transplant dialysis because of hyperoxaluria. Donor kidneys preserved on SCS are excluded, as well as kidneys retrieved after normothermic regional perfusion. Patients are asked informed consent by a qualified member of the research team while being waitlisted or if a donor kidney is offered to the patient. Eligibility criteria are assessed when a kidney is allocated to the recipient.

**Table 3:** In- and exclusion criteria.


KIDNEY RELATED	

**Inclusion**	**Exclusion**

– Kidney is preserved on HMP	– Kidney is preserved on static cold storage

– DCD kidney Maastricht type III, IV, V	– DCD kidney Maastricht type I, II

– Expanded DBD kidney, defined as: – Donor ≥60 years or donor 50–59 years with 2 out of the following risk factors: • History with hypertension • Death from a cerebrovascular stroke • Last creatinine higher than 133 μmol/l	– Dual kidney transplant

	– Kidney is retrieved after normothermic regional perfusion

	– Donor age is <18 years

**RECIPIENT RELATED**	

**Inclusion**	**Exclusion**

– Written informed consent	– Recipient age is <18 years

– Dialysis at the time of transplant	– Recipient is pre-emptive at time of transplant

– Standard immunosuppression regimen	– Recipient of a multi-organ transplant

	– Recipient virtual panel reactive antibodies ≥85%

	– Recipient for who it is agreed in advance that dialysis after transplant is required, such as in the context of hyperoxaluria


### 2.3 Randomization

The randomization is stratified according to oxygenated or non-oxygenated HMP because all DCD kidneys of a donor 50 years or older currently receive oxygenated HMP in the Netherlands. Donor kidneys are randomized in a 1:1 fashion using Alea software (Forms Vision bv) with a random block size varying between a total block size of 4 and 6. Randomization takes place after the recipient and donor kidney are deemed suitable for transplantation and the confirmation has been received that the donor kidney is transported on HMP. In exceptional cases, it may occur that the patient or donor kidney turns out to be unsuitable for transplantation. If the patient is unsuitable and the donor kidney is allocated to another eligible patient who wants to participate, the randomization arm is retained. If the kidney is randomized to another patient that does not meet the inclusion criteria or if the kidney is allocated to a patient in another center, the randomization will be withdrawn and not replaced. No one is blinded to treatment allocation because this is logistically not possible. Because all endpoints are objective and well-defined, any bias related to the absence of blinding is considered negligible.

### 2.4 Kidney retrieval and HMP procedure (both study groups)

All kidneys are retrieved by independent multi-organ donation retrieval teams according to national practice. In case of DCD donation, life-sustaining treatment is withdrawn, followed by circulatory arrest. After five minutes of obligatory ‘no touch’, the donor is transported to the operating room following super-rapid sterno-laporotomy and aortic flush with ice-cold University of Wisconsin (UW) solution. Consequently, kidneys are procured and immediately placed on HMP before transplantation. Two types of devices are used for HMP: the Kidney Assist-Transport (XVIVO, Göteborg, Sweden) and the LifePort (Organ Recovery Systems, Chicago, USA). The Kidney Assist-Transport also has a function of adding oxygen into the perfusion system at a rate of 100 ml/min. Therefore, all DCD donor kidneys 50 years or older are transported on the Kidney Assist-Transport. During HMP, a pulsatile flow of UW preservation solution is delivered at 1–4 degrees Celsius with a fixed pressure of 25 mmHg.

### 2.5 Investigational medical device (NMP group)

**Figure 2:**
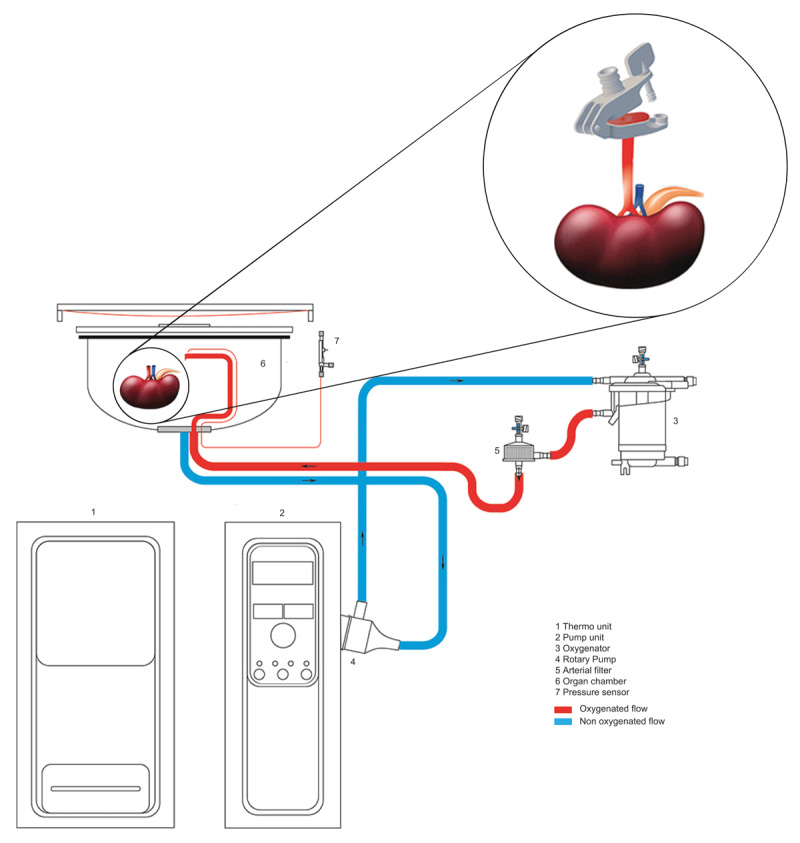
Perfusion circuit of the Kidney Assist.

The Kidney Assist (XVIVO, Göteborg, Sweden) is used for NMP, which is a pressure-controlled, CE marked medical device designed specifically for ex-vivo machine perfusion. The machine itself consists of a reservoir, a thermo unit and a pump unit with a display showing perfusion time, renal blood flow, pressure, intrarenal resistance and temperature of the perfusate. The disposable set consists of PVC tubing, a rotary pump and a membrane oxygenator with heat exchanger and an integrated arterial filter. The rotary pump is providing pulsatile perfusion at a speed of 60 beats per minute. The temperature of the perfusate is adjustable from 10 to 38 degrees Celsius. The disposable set also contains a sampling line for arterial and venous samples. ***Figure 2*** shows a graphical explanation of the Kidney Assist set up.

### 2.6 Preparation of the Kidney Assist (NMP group)

When the kidney arrives in the recipient hospital, the final approval of kidney suitability is made. Consequently, the Kidney Assist is prepared by connecting the disposable to the machine and fill it with all perfusate components. The composition of the perfusate is largely based on the Hosgood protocol and contains one unit of washed red blood cells which is cross-matched to the intended recipient [20]. As all deceased donor transplantations are ABO compatible in the Netherlands, the red blood cells are automatically also ABO compatible with the donor. Other components of the perfusate solution are presented in ***Table 4***. Three infusion pumps containing medication are also incorporated in the system and connected to the membrane oxygenator. After the system is de-aired, the pressure is set to 75 mmHg which is the normal mean arterial pressure in humans. The primed circuit is warmed up until 37 degrees and oxygenation with 100% O2 is started 15 minutes before reperfusion at a flow of 0.5 l/min. During the preparation of the Kidney Assist, the transplant surgeon disconnects the donor kidney from the HMP device under sterile conditions for back-table preparation. During back-table preparation, additional tissue is removed from the kidney and an arterial reconstruction is performed in case of vascular multiplicity. A biopsy is taken during benching of the kidney in both the intervention and control group. The renal artery with aortic patch is connected to a patch holder (***Figure 2***). The ureter is cannulated with a sterile 6 or 7 French catheter, depending on ureter size.

**Table 4:** Composition of perfusate used for NMP.


CONTENT	FINAL VOLUMES

1 unit of washed, cross-matched red blood cells	~275 ml

Albumin 20%	50 ml

Sterofundin® solution	1000 ml

Cefazolin	1 gram

Calcium gluconate 10%	10 ml

Sodium bicarbonate 8.4%	10–20 ml (10 ml at start of perfusion, then blood gas analysis: if pH < 7.35 add another 10 ml)

Mannitol 15%	20 ml (added after reperfusion)

Acetylcysteine	600 mg

Dexamethasone	8 mg

Heparin	2000 IU/100 gram donor kidney

**Infusions**:	

Pump 1: – Epoprostenol	80 microgram (40 microgram/h)

Pump 2: – Aminoplasmal 10% – Cernevit multivitamins – Insulin	50 ml (25 ml/h) 1 vial (0.5 vial/h) 100 IU (50 IU/h)

Pump 3: – Glucose 5%	14 ml (7 ml/h)


### 2.7 Perfusion of the kidney (NMP group)

Finally, the kidney is flushed and connected to the Kidney Assist, after which NMP is initiated. Mannitol is added immediately after reperfusion. Venous return from the renal vein passively drips back into the reservoir. During NMP, renal blood flow (in ml/min), pressure (in mmHg), intrarenal vascular resistance (in ml/min/mmHg) and temperature are continuously registered through the Kidney Assist. Urine output is monitored every 30 minutes and recirculated into the reservoir as a previous study found this provided a more physiologic acid-base stability [30]. Arterial and venous blood gas analyses are used to measure acid-base balance every 30 minutes. In case of acidosis (defined as pH <7.35), extra bicarbonate is added into the perfusion circuit to correct the pH. Kidney weight is measured before and after NMP and photos of renal macroscopy are taken hourly. Biopsies, perfusate and urine samples are taken on predefined time points (***Table 2***). Flow and oxygenation of the renal microcirculation are monitored after 15, 75 and 105 minutes with the MoorO2Flo laser speckle camera (Moor instruments, Devon, UK). The pressure is kept constant during NMP. A surgeon will be present during the whole NMP procedure for trouble-shooting and to switch immediately to static cold storage in case of mechanical problems. The recipient operation is started approximately 60 minutes after initiation of NMP. After 120 minutes NMP, the donor kidney is taken of the machine and flushed with UW solution. Consequently, the donor kidney is taken to the operating room of the recipient for immediate implantation or shortly kept on static cold storage in case immediate transplantation is logistically not possible.

### 2.8 Transplantation procedure and follow-up (both groups)

The kidney transplantation is performed using the standard technique in the right or left iliac fossa, depending on preference of the transplant surgeon. After exposure of the external iliac artery and vein, the venous anastomosis is performed followed by the arterial anastomosis. Both the arterial and venous anastomosis are performed in an end-to-side fashion. The arterial anastomosis is made with the external iliac artery or common iliac artery and the venous anastomosis with the external iliac vein or common iliac vein. For the ureter, an extra-vesical anastomosis is performed which is protected with an external splint or double J stent. In the control group, a biopsy is obtained after the ureterovesical anastomosis to compare with the NMP reperfusion biopsies. Based on our inclusion criteria, all transplant recipients receive standard immunosuppression. During hospitalization, serum laboratory values including kidney function are measured daily according to standard of care. Two extra cell-save tubes are collected on day 1 to 6 for measurements of donor-derived cell-free DNA as a surrogate marker of ischemia-reperfusion injury [27]. All patients receive regular check-ups in the first year after transplant according to standard of care.

### 2.9 Statistical analysis

All analyses of the primary and secondary endpoints will adhere to the intention-to-treat principle. Furthermore, the primary and secondary outcomes will also be analyzed per protocol. Lastly, a secondary analysis will compare clinical outcomes of NMP kidneys to their contralateral kidneys if they are also transplanted.

#### 2.9.1 Sample size calculation

The present study is powered to detect a difference in the proportion of patients experiencing immediate function after transplant between the 2 study groups. In our pilot study (POSEIDON study), the proportion of kidney-related immediate function in the NMP group was 70% compared to 40% in the control group [21]. Based on the results from this pilot study, we require a sample size of 40 patients per group with a 2-sided α of 5% and power (1-β) of 80%. With a 1:1 randomization, this leads to a total sample size of 80 patients. Because drop-out is very unlikely due to our early primary endpoint in the first week after transplant, we decided not to include extra patients.

#### 2.9.2 Analysis of primary and secondary outcomes

The analysis of the primary endpoint and all secondary endpoints will be described in detail in a full statistical analysis plan. The statistical analysis plan will be drawn up prior to the end of study. This section will only describe the main analyses. The primary outcome (i.e. immediate function) is presented as numbers and percentages and groups are compared using the chi-square test. Consequently, a logistic regression analysis adjusting for oxygenated hypothermic machine perfusion, total cold ischemic time and donor age is performed. No additional variables are taken into account to prevent overfitting. All categorical secondary endpoints are compared with chi-square tests or fisher’s exact test. Continuous secondary endpoints are compared with Mann-Whitney U test. eGFR trajectory until 1 year after transplantation is analyzed using a linear mixed model with an unstructured correlation matrix. Survival outcomes are plotted with Kaplan-Meier curves and compared with log-rank tests. For comparisons made with the contralateral kidneys, paired analyses are performed. For all statistical tests, a two-sided p-value <0.05 is considered statistically significant.

## 3. Ethics and dissemination

### 3.1 Ethical approval and monitoring

The protocol of this study and all study documents are approved by the Medical Ethical Committee of Erasmus Medical Center (APOLLO study, MEC 2020-0366). Amendments made to the study protocol require ethical review. Insurance for study participants is covered under the Erasmus Medical Center clinical trial policy. The study will be audited by a qualified monitor every 4–6 months to review whether study procedures are performed in accordance with Good Clinical Practice. All data is collected prospectively and recorded anonymously in electronic case report forms using Castor electronic data capture. Patient data and tissue samples will be stored for 15 years.

### 3.2 Risks and reporting of (serious) adverse events

Because of the normothermic nature of the perfusion, failure of the process leads to warm ischemia. During our previous pilot study, no failure occurred [21]. If a failure would occur, a highly trained transplant surgeon is present at all time during the perfusion to switch to SCS immediately. Therefore, extra risk of the NMP procedure is deemed small. Renal biopsies are obtained hourly during machine perfusion. In the control group, one renal biopsy is obtained during benching and one after ureter anastomosis. In exceptional cases, a biopsy could lead to postoperative bleeding. This risk is deemed small because the biopsy punctures are closed with sutures after NMP. Adverse events and serious adverse events are scored according to the Common Terminology Criteria for Adverse Events (CTCAE, version 4.0) until 14 days after transplantation. Serious adverse events will be reported within 7 days of knowledge to the medical ethical committee of Erasmus Medical Center.

### 3.3 Dissemination of study results

Results of this study will be submitted to peer-reviewed journals and will be presented at national and international conferences

## 4. Discussion

This is the first randomized, controlled trial to compare 120 minutes of additional, end-ischemic NMP to HMP alone. The results of this study can help identify whether the addition of a short period of NMP is superior to HMP alone for suboptimal kidney grafts. We chose to include only suboptimal kidney grafts as these are expected to benefit the most from potentially better preservation strategies because of the more pronounced ischemia-reperfusion injury.

The results from our study can help further develop NMP as an assessment tool by identification of biomarkers or perfusion characteristics that correlate well with transplant outcomes. Due to the lack of clinical studies, no urine or perfusate biomarkers have been investigated or validated in large cohorts of kidney transplants [31]. Hosgood et al. defined a simple kidney assessment score based on urine production, flow, and macroscopic appearance during NMP [29]. Usage of this score on a small number of declined donor kidneys resulted in more transplantable kidneys without any PNF [32]. The present RCT can help define more precise viability criteria because of the larger sample size compared to previously published small clinical studies. Identification of these biomarkers is of paramount importance to objectively measure graft quality and graft function during NMP. These biomarkers can be used in the future to define viability criteria for declined donor kidneys to be accepted for transplantation, which may increase the amount of transplantable donor kidneys. This concept has proven to work in the case of liver transplantation, as the use of combined HMP and NMP to assess declined donor livers resulted in a 20% increase in the number of deceased donor liver transplantations [33]. Lastly, the high number of DCD kidneys being transplanted is accompanied by higher numbers of PNF [34]. The possibility of viability assessment pre-transplant may help decrease the numbers of PNF kidneys being transplanted.
